# Health care curricula in multicultural societies

**DOI:** 10.5116/ijme.5a7e.bd17

**Published:** 2018-02-21

**Authors:** Esperanza Diaz, Bernadette N. Kumar

**Affiliations:** 1 Department of Global Public Health and Primary Care, University of Bergen, Norway; 2 Norwegian Center for Migration and Minority Health, Norwegian Institute of Public Health, Oslo, Norway

**Keywords:** Health care, curricula, multicultural societies, Norway

## Introduction

Globalisation has led to increasing diversity worldwide. The rapid rise in the number of international migrants over the last 15 years has been unprecedented and currently accounts for more than 10% of the European and 16% of the Norwegian populations. Most countries in Europe attempt to provide equitable health care services to their citizens regardless of their ethnicity, religion, country of origin and other characteristics.^1^ Still, a large body of literature describes challenges in providing healthcare for multicultural populations for doctors and other health care professionals.^2^^,^^3^ Furthermore, European health professionals are not at ease providing adequate health care in multicultural societies and in many countries they are still to receive systematic training to tackle this new and complex situation.^4^

Intercultural challenges are often attributed to the immigrant patients alone. However, the responsibility of health professionals in initiating, maintaining or inadequately tackling these challenges cannot be ignored. Although international recommendations to improve cross-cultural care exist,^5^ health professionals still perform differently in diagnostic procedures undertaken,^6^^,^^7^ number of consultations needed for referral to secondary care,^8^^,^^9^ specificity of diagnoses provided or treatments given^10^^,^^11^ for immigrant patients compared to non-immigrants. Furthermore, immigrants from low and middle-income countries in Europe seem to be less satisfied with health care services than the majority population.^12^^,^^13^

The so-called refuge-crises in 2015 actualised the necessity of dealing with intercultural consultations as a part of everyday work for healthcare professionals. Not surprisingly the authors have frequently been contacted to lecture about migration and health for different audiences. This demand responds to self-perception of lack of knowledge, skills or competencies necessary to give equitable health care to a growing number of immigrant patients.^14^^,^^15^

This paper aims to present and argue the need for a profound change in the healthcare curricula by describing our observations and experiences within medical education.

### Teaching undergraduate students

We will first describe the situation of undergraduate medical students at the end of their education. At the beginning of our lessons, we often explore their experiences and knowledge to ensure that our teaching builds from their current state. Typical questions are:

  ·     What health care entitlements can immigrants and refugees receive at the different stages of their life in Norway?

  ·     Are there specific risk factors or diseases to be kept in mind, in consultations with patients of migrant origin?

  ·     Do we talk about culture with patients? Should we talk about culture at all? How to have effective consultations with translators?

  ·     Should we culturally adapt healthcare interventions for specific groups or choose a diversity-sensitive approach that accommodates migrants?

As per our observations, few students can answer these or similar questions despite their clinical encounters with patients with immigrant background through their clinical rotations during several semesters. Furthermore, they can seldom refer to senior clinicians who have been good role models for cross-cultural consultations.

Moreover, the simple exercise of describing one’s own ethnic and cultural background is often an eye-opener for many students and practitioners. It makes them realise that it is far too easy to describe another persons’ background by the different colour of their skin, their different clothes or because they speak with an accent. It is relatively easy for immigrant students or for Norwegians, who are proud of their regions and local dialects, to describe themselves if they have moved within the country, but the task is far more challenging for those who have never moved out of these local communities.

### Courses for health professionals working in care for the elderly

Healthcare professionals working for the elderly have historically dealt with few immigrants in Norway since most migrants are young at arrival. During two post-graduate talks in 2015 and 2017 among doctors and nurses working at nursing homes and home care for the elderly, the first author systematically asked the participants about their experience with immigrant patients. Most health professionals had patients with various immigrant backgrounds and faced daily challenges in the care of these patients regarding not only language and communication, but also with dealing with family members, intimate care, situations around death and different expectations for care of the patient and in the process of decision making. A clear minority of doctors considered that immigrant patients received the same quality of care compared to Norwegian-born patients. Despite the use of translators, personnel with immigrant background and family members to overcome language and cultural barriers, health personnel expressed the need for better access to professional translators and further courses regarding cross-cultural consultations were widely requested.

## Discussion

Most health professionals’ curricula in Norway and Europe build upon “patient-centered care” concepts and theories, hoping that being attentive to the persons’ background and needs, and carefully listening to his/her symptoms and stories will be enough to meet the patient “where he/she is.” While this is true for many consultations, it ignores challenges specific to many immigrants such as cultural, linguistic and personal perspectives as well as system-related perspectives particular to cross-cultural encounters, as thoroughly discussed by Saha et al.^16^

In our first example, students were surprised when they reflected upon their own culture and ethnicity. A fairly common human trait is to believe that we are cultureless and thus the starting point or reference standard for measuring others. This phenomenon often called ethnocentrism can at its worst, have consequences like the creation of new diseases; “Drapetomania”, a mental illness described in 1851 as the addiction of slaves to attempt escaping slavery. On the other hand, culturalism, a theoretical approach emphasizing the significance of culture as a determinant of individual behaviour, can also be dangerous, leading to stereotyping, not leaving room for the uniqueness of a person or impairing dialogue between cultures. Students and health personnel should, therefore, be aware that every individual has a culture, but that birth does not predetermine it. Indeed, the task is not easy: the health professional must understand the role culture plays for him or herself and the individual patient, but not to overplay it. Also, health professionals play a key role in gradually helping migrants to learn the culture of the health system in their new country.

Our experiences regarding health professionals concur with international literature.^17^ One-fourth of resident physicians participants of a survey in USA were unable to provide specific components of cross-cultural care, including caring for newly arrived immigrants, patients with health beliefs at odds with Western medicine, and patients whose religious beliefs affected treatment. In addition, the physicians lacked the knowledge and skills to identify relevant cultural practises that affected medical care. In contrast, only 1-2% reported that they were unable to treat clinical conditions or perform procedures common to their specialties.18 In Norway, a recently published study among General Practitioners showed a lack of specific strategies to deal with cross-cultural consultations.^15^

Earlier reports from the University of Oslo, Norway showed that although migration and health as a subject was covered for both doctors and nurses, it was only a few optional hours at pre-graduate level, often lacking learning objectives as well as adequate and relevant clinical practice assessments.^19^^,^^20^ Most of the students reported a lack of competence in this area at the end of the study. Documentation of the extent of teaching on migration and health at other undergraduate academic sites in Norway is limited, but it is highly unlikely that the situation is better elsewhere in the country.

Cultural competence in its various definitions and forms is nowadays part of the curriculum of some English-speaking countries with a longstanding history of migration. Two recent reviews of the literature assessing the effects of cultural competence education for health professionals show positive, albeit low-quality-evidence, improvements on patient-related and healthcare organisation outcomes.^21^^,^^22^ According to a recent Norwegian study, health professionals with cultural competence skills more often keep an open mind, clear up misunderstandings, ask pertinent questions regarding ethnic minority patients’ needs, perceptions and wishes and develop inner confidence reflected as reliability and trustworthiness.^23^

However, isolated one-time interventions to improve cultural awareness and competence are like a flash in the pan. Practical experience and guidance when caring for migrant patients must be addressed in the long term.^24^ Existing discrepancies between the formal curriculum, the informal curriculum, and what is delivered through role models in everyday clinical work, or the hidden curriculum, are specially important for migration and health as a discipline and have to be targeted transversally through the curriculum.^25^

To bridge the gap between theory and practice and attain equity in health care, it is, therefore, necessary to ensure adequate cultural awareness, knowledge on migration and health and cross-cultural competencies among health care professionals. The advent of rapidly increasing migration to Europe and the early transfer of refugees to mainstream care, pose indeed an urgent need to include this subject as a transversal element, grounded on clinically relevant practice, in pre-graduate healthcare curricula. We therefore argue that cross-cultural competence, in particular responding to diversity, should be mandatory both in theory and practice.

### Acknowledgements

We thank all the students and practitioners for sharing their experiences with us.

### Conflict of Interest

The authors declare that they have no conflict of interest.
